# Etiology and outcomes of convulsive status epilepticus in children

**DOI:** 10.12669/pjms.35.3.120

**Published:** 2019

**Authors:** Muhammad Uzair, Asif Ibrahim, Faisal Zafar, Tipu Sultan

**Affiliations:** 1 *Dr. Muhammad Uzair MBBS, FCPS (Pediatrics), Fellow Pediatrics Neurology, Department of Pediatric Neurology, Children Hospital Lahore, Pakistan*; 2 *Dr. Asif Ibrahim MBBS, FCPS (Pediatrics), Fellow Pediatrics Neurology. , Department of Pediatric Neurology, Children Hospital Lahore, Pakistan*; 3 *Dr. Faisal Zafar MBBS, FCPS (Pediatrics), Fellow Pediatrics Neurology. , Department of Pediatric Neurology, Children Hospital Lahore, Pakistan*; 4 *Dr. Tipu Sultan MBBS, FCPS (Pediatrics), MRCPCH (UK). Associate Professor, Department of Pediatric Neurology, Children Hospital Lahore, Pakistan*

**Keywords:** Etiology, Outcomes, Status Epilepticus, Children

## Abstract

**Objective:**

The study aimed to ascertain different causes and outcomes of convulsive status epilepticus in children.

**Methods:**

From January 2018 to June 2018, seventy three patients who presented with status epilepticus were studied. Data were recorded with the help of a pre-formed performa. Etiological factors and outcomes in terms of recovery, morbidity and mortality were studied.

**Results:**

Out of 73 children, forty one (56%) were males and 32(44%) were females with median age of 1.09±0.27 years. Etiologies were acute symptomatic 25(34%), febrile 19(26%), progressive encephalopathy 10(14%), remote symptomatic 10(14%) and idiopathic 7 (9%) with p-value 0.005. Status epilepticus was controlled within one hour in 42(57%), within 1-6 hours in 21(29%) and more than 6 hours in 10(14%) patients with p-value 0.027. During hospitals stay, twenty one (29%) patients recovered completely, seizure recurred in 12(16%), Twelve (16%) became mentally retarded, Twelve (16%) developed mental retardation along with seizures and 16(22%) died. Eight (10.9%) deaths were attributed to acute symptomatic etiology with p-value less than 0.001.

**Conclusion:**

This study concluded that acute symptomatic etiology was more common cause of status epilepticus as compared to other etiologies and it is associated with poorer outcomes as compared to other etiologies.

## INTRODUCTION

Status epilepticus is considered a very common neurological disease which usually presents as an emergency thus requires immediate steps for prevention of permanent damage to the brain tissues.^1^

Prevalence is usually high in extreme of ages i.e. in children and elderly adults, but it can occur almost at any age in the form of prolonged seizures.^1^,^2^ There are multiple conditions in which status epilepticus can present in children, infections, hypoxia, head trauma, cerebral malformations and patients with previously established epilepsy.^3^ The major predictor of outcomes following convulsive status epilepticus is etiology.^4^

Incidence of status epilepticus in children is reported to be 18 per 100000 per annum.^5^ Studies have shown that status epilepticus is extensively associated with high rates of mortality and morbidity but not enough data is present regarding the fact that whether these severe outcomes are actually caused by status epilepticus itself or if these are the result of other contributing factors like, treatment offered or the age of the patient.^6^ Previously reported adverse outcomes of status epilepticus include, permanent neurological damage, subsequent epilepsy, cognitive impairment and hippocampal injury.^4^ Status epilepticus by its operational definition is a continuous seizure that continues for more than five minutes without arresting phase or returning to normal or more than one seizure within a period of five minutes.^7^ Classification of status epilepticus is as diverse as the types of epileptic seizures, when it is based on clinical settings.^8^ Practically status epilepticus has following classification, convulsive or non convulsive.

In current study our focus was on convulsive status epilepticus. Importance of this study can be recognized from the fact that study of etiology and possible outcomes in terms of mortality and morbidity of the patients could help in making recommendations for the clinicians. It could also help in better understanding the pattern of the status epilepticus. Rationale of this study was that no much data regarding the etiology and outcomes of the status epilepticus is available especially in this community of the world. In this study by assessing the possible causes of status epilepticus and its outcomes in children, we can make better decisions for management of status epilepticus.

## METHODS

It is a hospital based retrospective cross sectional study performed in department of Pediatrics Neurology in Children Hospital and Institute of Child Health, Lahore from January 2018 to June 2018. Ethical approval for this study was obtained from the hospital ethics committee. During this period a total number of 73 patients presented to the emergency with status epilepticus. Inclusion was based upon patients aged 1 to 14 years of age who presented with clinical or subclinical seizures and belonging to either gender. All those patients aged more than 14, presented with seizures not included in the definition of status epilepticus or patients with developmental abnormalities, patients unwilling to participate in the study or patients diagnosed to have degenerative disease of brain. Reference for this study was taken from a previous article by B. Tabarki et al.^9^ Sampling technique was non probability consecutive sampling technique. Operational definition of status epilepticus used in this study was that a seizure continuous in character for more than five minutes or multiple seizures occurring consecutively during which patients were unable to regain consciousness over a time period of 30 minutes. Demographic and baseline data was recorded with the help of a performed performa. Variables included age, sex, type of status epilepticus, cause, duration of convulsions, duration of unconsciousness, precipitating factors, EEG and number of anti epileptic drugs required to control the seizures, history of convulsions and fever, any complications occurred and ultimate survival or death. Baseline investigation were also carried out including, complete blood count, blood glucose levels, serum electrolytes, blood urea and creatinine, urinalysis, lumbar puncture, MRI and EEG were performed. Data thus collected was subjected to statistical analysis with the help of computer software SPSS version 23. Chi square test was applied and p value less than or equal to 0.005 was considered significant.

**Table-I:** Baseline Data.

Variable	Value
Median age, years (SD)	1.09±0.27
Gender, Male/female n(%)	41 (56)/32(44) (total n=73)
Prior history of seizures, n (%)	24 (32.8)
Mean time between onset of seizures and hospital presentation, min	54.9±25.4
Pre-hospital treatment, n (%)	5 (6.8)
Seizure Type, n (%)	
Simple partial	12 (16.4)
Complex partial	4 (5.5)
Partial with Secondary generalization n(%)	15 (20.5)
Unclassifiable	7 (9.6)
Generalized	35 (47.9)

## RESULTS

We selected seventy three children of which 41 were males and 32 were females. Mean age of all the children was 1.09±0.27 years. Previous history of seizures was positive in 32.8%(24) of the children. Mean time of presentation at the hospital after the onset of seizures was 54.9±25.4 minutes. Only five children received some treatment prior to their arrival at the hospital. Generalized seizures were seen 47.9%(35) of the patients, 16.4%(12) had simple partial seizures, 5.5% had complex partial seizures, 20.5%(15) had partial seizure with secondary complications and the seizures in remaining 9.6%(7) of the patients were unclassifiable on the basis of history. Acute symptomatic and febrile etiology was more common in children below one year of age, whereas remote symptomatic was more common in older children (p=0.005). Most of the acute symptomatic seizures lasted for one to six hours whereas majority of the febrile fits lasted for less than one hour (p=0.027). Almost 40% of the patients had generalized seizures with which had acute symptomatic and febrile cause (p=0.043). Other etiologies had no specific association with any specific type of the seizure.

**Table-II:** Relationship between etiology of status epilepticus and the age of occurrence.

Etiology	≤ 1 year	>1 year	p-value
Acute symptomatic, n (%)	19 (26.0)	6 (8.2)	0.005
Febrile, n (%)	18 (24.6)	3 (4.1)	
Progressive encephalopathy, n (%)	6 (8.2)	4 (5.5)	
Remote symptomatic, n (%)	2 (2.7)	8 (10.9)	
Idiopathic, n (%)	5 (6.8)	2 (2.7)	

**Table-III:** Etiology vs duration of status epilepticus.

Etiology	<I hour	1-6 hours	>6 hours	p-value
Acute symptomatic, n (%)	8 (10.9)	13 (17.8)	4 (5.5)	0.027
Febrile, n (%)	14 (19.2)	5 (6.8)	2 (2.7)	
Progressive encephalopathy, n (%)	6 (8.2)	1 (1.4)	3 (4.1)	
Remote symptomatic, n (%)	7 (9.6)	2 (2.7)	1 (1.4)	
Idiopathic, n (%)	7 (9.6)	0 (0)	0 (0)	

**Table-IV:** Etiology vs type of seizures.

Etiology	Simple partial	Complex partial	Partial with secondary generalization	Unclassifiable	Generalized	p-value
Acute symptomatic, n (%)	5 (6.8)	1 (1.4)	2 (2.7)	1 (1.4)	16 (21.9)	0.043
Febrile, n (%)	3 (4.1)	1 (1.4)	2 (2.7)	1 (1.4)	14 (19.2)	
Progressive encephalopathy, n (%)	1 (1.4)	0 (0)	5 (6.8)	1 (1.4)	3 (4.1)	
Remote symptomatic, n (%)	1 (1.4)	1 (1.4)	4 (5.5)	3 (4.1)	1 (1.4)	
Idiopathic, n (%)	2 (2.7)	1 (1.4)	2 (2.7)	1 (1.4)	1 (1.4)	

Outcome was poor in the acute symptomatic group with 10.9%(8) deaths, 8.2%(6) recoveries, 6.8% secondary seizures, 4.1%(3) mental retardation and 4.1%(3) mental retardation along with secondary seizures. Febrile group had best outcome with 13.7% recoveries. Remote symptomatic group had worst outcome with 9.6% having mental retardation along with secondary seizures, 4.1%(3) having mental retardation alone and no recovery at all (p<0.001).

## DISCUSSION

Results of our study suggest that etiologies are directly affecting the outcomes of status epilepticus. Previously in a systemic review of different studies, was performed in which observation was made that outcome of convulsive status epilepticus was affected by the causative factor of SE. It was also suggested in that systemic review that current data is insufficient to demonstrate the relation of age of onset of status epilepticus, treatment provided and duration of the seizures with the possible outcome.^10^

In a study it was concluded that convulsive status epilepticus in childhood differs from the one that occurs during the old age. As far as the etiology was considered febrile seizures comprised the highest number and it was reported that acute bacterial meningitis might be the reason for the first episode of convulsive status epilepticus in children.^11^

It has been reported that age is a significant factor in determination of onset of status epilepticus as it occurs in extremes of age and even during childhood there is remarkable difference between its incidence among younger and older children. Fever has been reported to be the number one cause of status epilepticus. Forty percent of the children presenting with SE have previous neurological abnormality and fifteen percent have history of epilepsy. Outcome is related to the etiology.^12^

**Table-V:** Mortality and morbidity in status epilepticus.

Etiology	Death	Complete Recovery	Seizures Recurrence	Mental retardation	Mental retardation along with seizures	p-value
Acute symptomatic, n (%)	8 (10.9)	6 (8.2)	5 (6.8)	3 (4.1)	3 (4.1)	<0.001
Febrile, n (%)	2 (2.7)	10 (13.7)	4 (5.5)	3 (4.1)	2 (2.7)	
Progressive encephalopathy, n (%)	5 (6.8)	0 (0)	3 (4.1)	2 (2.7)	0 (0)	
Remote symptomatic, n (%)	0 (0)	0 (0)	0 (0)	3 (4.1)	7 (9.6)	
Idiopathic, n (%)	1 (1.4)	5 (6.8)	0 (0)	1 (1.4)	0 (0)	

In a previously performed study seventy children were included in the study presenting with SE and among these children no mortality occurred. None of the children had previous neurological damage. After admission, just one child developed novel neurological abnormality. Five percent of the patients developed new epilepsy.^13^ In a study where observation was made among patients of all ages suffering from status epilepticus, they suggested that poor outcomes are more common in older age and also their loss of consciousness is also largely associated with higher incidence of mortality. It also showed that those patients who survived their first attack of status epilepticus had lower mortality and morbidity suggesting that etiology is the main determinant of outcome instead of SE itself.^14^

Acute symptomatic seizures and increased length of hospital stay after admission are also associated with higher rates of mortality as suggested by a previous analysis.^15^ The most adverse outcome i.e. mortality was related to the age of the patients, cause of status epilepticus and EEG findings that were taken after admission.^16^ Fever has been reported as the most common cause of SE and it also affects the outcome unlike other factors like age, sex.^17^,^18^ Status epilepticus when occurs in neonates, it is highly associated with post neonatal epilepsy and neurological damage in neonates.^19^

## CONCLUSION

Acute symptomatic etiology was more common cause of status epilepticus as compared to other etiologies and that it is associated with poorer outcomes as compared to other etiologies.

### Authors’ Contribution

**MU & TS:** conceived, designed and writing / editing of manuscript.

**AI & FZ:** data collection and manuscript writing.

**AI:** Statistical analysis.

**TS:** Review, editing and final approval of manuscript.
